# Epidemiology of frequent attenders: a 3-year historic cohort study comparing attendance, morbidity and prescriptions of one-year and persistent frequent attenders

**DOI:** 10.1186/1471-2458-9-36

**Published:** 2009-01-24

**Authors:** Frans ThM Smits, Henk J Brouwer, Gerben ter Riet, Henk CP van Weert

**Affiliations:** 1 Division of Clinical Methods and Public Health, Department of General Practice Academic Medical Centre, University of Amsterdam, PO Box 22700, 1100 DE, Amsterdam, The Netherlands; 2 Medical Health Centre Reigersbos, Ravenswaaipad 110, 1107EG, Amsterdam, The Netherlands; 3 Horten-Zentrum, University of Zürich, Zürich, Switzerland

## Abstract

**Background:**

General Practitioners spend a disproportionate amount of time on frequent attenders. So far, trials on the effect of interventions on frequent attenders have shown negative results. However, these trials were conducted in short-term frequent attenders. It would be more reasonable to target intervention at persistent frequent attenders. Typical characteristics of persistent frequent attenders, as opposed to 1-year frequent attenders and non-frequent attenders, may generate hypotheses regarding modifiable factors on which new randomized trials may be designed.

**Methods:**

We used the data of all 28,860 adult patients from 5 primary healthcare centers. Frequent attenders were patients whose attendance rate ranked in the (age and sex adjusted) top 10 percent during 1 year (1-year frequent attenders) or 3 years (persistent frequent attenders). All other patients on the register over the 3-year period were referred to as non-frequent attenders. The lists of medical problems coded by the GP using the International Classification of Primary Care (ICPC) were used to assess morbidity.

First, we determined which proportion of 1-year frequent attenders was still a frequent attender during the next two consecutive years and calculated the GPs' workload for these patients. Second, we compared morbidity and number of prescriptions for non-frequent attenders, 1-year frequent attenders and persistent frequent attenders.

**Results:**

Of all 1-year frequent attenders, 15.4% became a persistent frequent attender equal to 1.6% of all patients. The 1-year frequent attenders (3,045; 10.6%) were responsible for 39% of the face-to-face consultations; the 470 patients who would become persistent frequent attenders (1.6%) were responsible for 8% of all consultations in 2003. Persistent frequent attenders presented more social problems, more psychiatric problems and medically unexplained physical symptoms, but also more chronic somatic diseases (especially diabetes). They received more prescriptions for psychotropic medication.

**Conclusion:**

One out of every seven 1-year-frequent attenders (15.4%) becomes a persistent frequent attender. Compared with non-frequent attenders, and 1-year frequent attenders, persistent frequent attenders consume more health care and are diagnosed not only with more somatic diseases but especially more social problems, psychiatric problems and medically unexplained physical symptoms.

## Background

General practitioners (GP) spend a large part of their time on a small proportion of their patients. It is estimated that about 80% of a GP's clinical work is spent on 20% of their patient [1]. Because a proportional threshold definition allows meaningful comparison between practices, periods and countries most studies define frequent attendance as an age and sex-adjusted attendance rate ranking in the top 10 centile within a time frame of one year (1-year-frequent attenders) [2,3].

Systematic reviews show that these 1-year frequent attenders are more likely to suffer from physical and psychiatric illness, social difficulties and emotional distress [2,4,5]. High attendance rates are also found for patients with medically unexplained somatic symptoms, health anxiety and perceived poor health [5-7]. In addition, frequent attendance may be a sign of inappropriate consultation behaviour [8-11].

At this point, we should ask the question whether or not it is possible to treat the morbidity of frequent attenders and reduce their attendance rates? Trials on the effect of (mainly psychiatric) interventions have shown conflicting results [12]. No study has shown convincing evidence that any intervention improves the quality of life or morbidity of frequent attenders in primary care, although there is some evidence that an effect might exist in a subgroup of frequent attenders – that of depressed patients. There is no evidence to suggest that the utilization of health care by frequent attenders can be influenced. The only trials that showed positive effects were with patients who were frequent attenders over a period of two years; all others used a time frame of one year [13,14]. This means that these studies may have targeted the wrong group of transient frequent attenders.

Until now most research on frequent attendance has been cross-sectional and used 1-year attendance rates. The few longitudinal studies conducted showed regression of attendance to the mean in the longer run, with only 20–30% of frequent attenders continuing to attend frequently in the following year [15-17]. However, these studies on persistent frequent attendance used different definitions of frequent attenders and lacked the power to detect differences in morbidity between transient and persistent frequent attenders. Readily available indicators from GPs electronic medical record performed modestly in predicting future persisting attendance[18].

This study presents the results of a historic 3-year cohort study on 28,860 adult patients in a longitudinal primary care database. Our first objective was to determine the proportion of 1-year frequent attenders who remain a frequent attender during two consecutive years and to calculate the GP workload for non-frequent attenders, 1-year frequent attenders, and persistent frequent attenders. Secondly, we wanted to determine whether and how persistent frequent attenders differ from 1-year frequent attenders and normal attenders.

## Methods

### Patient population

Five primary healthcare centres in Amsterdam provided data for this study. These centres participate in the GP-based continuous morbidity registration network of the Department of General Practice at the Academic Medical Centre of the University of Amsterdam. The studied patients have a lower socio-economic level, are of more non-western descent and are slightly younger than the Dutch population. In this GP network, electronic medical record data are extracted for research purposes. The participating GPs use a problem oriented registration method. For this study we used the following data: the numbers of face-to-face GP consultations, the lists of patients' current medical problems as registered and coded by the GPs using the ICPC, the number of a selection of prescriptions for all enlisted patients from 1 January 2003 through 31 December 2005.

The study was conducted according to the Dutch legislation on data protection (Ministry of Justice, the Netherlands).

### Selection of 1-year frequent attenders, persistent frequent attenders and non-frequent attenders

Frequent attenders were defined as those patients whose attendance rate ranked nearest to the top 10th centile of their sex and age group (15–30 years; 31–45 years; 46–60 years; 61 years+) [2,3]. Frequent attenders were determined for each of the years 2003, 2004 and 2005. As a starting point, we took the 1-year frequent attenders for the year 2003. We defined persistent frequent attenders as those patients who continued to be a frequent attender over the three year period. Patients who were never a frequent attender in the three year study period (non-frequent attenders) were used as a reference group. We compared the three selections. Patients younger than 15 years were excluded, because their consultations often involved the parents as well as the patient. A multivariable analysis was performed to check for selective loss to follow up.

### Attendance

Only face-to-face consultations with GPs (consultations in the surgery and house-calls) were included. Consultations with other practice staff were excluded because these contacts are mostly initiated by the GP and relate mostly to the monitoring of chronic diseases. We determined the mean number of consultations per age and sex group for the three groups of patients.

### Morbidity

In the problem oriented approach to medical record keeping, patients can have a list of current medical problems (problem list). Different from the definition used in the United Kingdom, in the Netherlands a current medical problem is defined by the GP as: any medical problem (disease or complaint) which needs continual medical attention or monitoring; any complaint or disease presented to the GP that has lasted more than 6 months.

Every problem on this list was coded by the GPs using the ICPC [19]. Please see the appendix (Additional file 1) for a list of the selected ICPC-codes.

The data from these problem lists were extracted at the end of 2003 and the end of 2005. The numerator in the prevalence calculations was the number of enlisted patients with a certain current problem at the end of the two periods. Thus the prevalence of each medical problem was calculated for 1-year frequent attenders at the end of the first year, for persistent and non-frequent attenders at the end of the third year. Prevalences were calculated for that subset of morbidity in which, according to the literature, frequent attenders differ most from normal attenders: diabetes mellitus, chronic cardiovascular disease, chronic respiratory disease, feelings of anxiety, feelings of depression, addictive behaviour, other psychological/psychiatric codes, all social problems and medically unexplained physical symptoms (MUPS) [2,4]. MUPS were defined according to Robbins et al. and had to comply with the definition of the problem list [20].

We determined the total number of registered medical problems as indicator of overall morbidity for the one and three year periods.

### Prescribed medication

The yearly number of prescriptions for each patient was calculated for the following: antibiotics, analgesics, anxiolytics, hypnotics, and antidepressants. We present the average number of prescriptions of these 5 groups of medications in non-frequent attenders, 1-year frequent attenders and persistent frequent attenders.

### Statistical analysis

SPSS 14.0 for windows was used for the statistical analysis. Differences between patient groups were analysed using X2 test. Statistical significance was set at P < 0.05. After checks for errors and consistency, we assessed the potential for selection bias due to loss to follow-up and death.

Text box 1 (Additional file 2) gives a description of our approach. Statistical analyses were performed in Stata (version 9.2).

## Results

### 1-year frequent attenders, persistent frequent attenders and GP-workload

**Figure 1:**
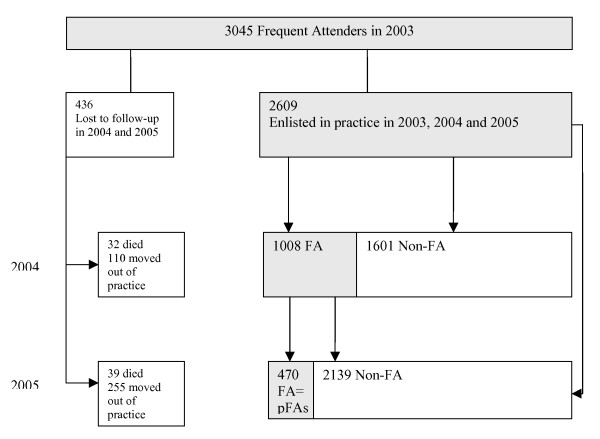
**Flow diagram: Persistence of Frequent Attendance**.

Of all 3,045 frequent attenders in 2003, 436 were lost to follow-up because they had died (71) or moved out of the practice (365) before December 31, 2005. A multivariable analysis showed (virtually) no signs of selective loss to follow up for moving out of the practice or for death (see Additional file 2). Of the 2,609 frequent attenders in 2003 who could be followed for three years, 1,008 were also found to be a frequent attender in 2004, while 470 continued to be a frequent attender in 2004 and 2005 and were a persistent frequent attender according to our definition. These persistent frequent attenders comprised 1.6% of all enlisted patients of 15 years and older in 2003 and 15.4% of all 1-year frequent attenders in 2003 (see Fig. 1). Compared with 1-year frequent attenders, persistent frequent attenders are slightly older. The percentage of patients over the age of 65 years changed from 12.5% to 15.3%, the percentage of patients in the age group 45–64 years changed from 26.6% to 34% and the percentage at 15–44 years decreased from 60.9% to 50.6%.

The number of yearly consultations varied substantially according to age. In 2003, the mean number of consultations of non-frequent attenders (n = 19,120), 1-year frequent attenders (n = 2,609) and persistent frequent attenders (n = 470) were 1.4, 7.8 and 10.2 respectively (see Table 1).

**Table 1:** Number of GP-consultations per age group for non-frequent attenders, 1-year frequent attenders and persistent frequent attenders in 2003.

	Non-Fas^1^	1 yFAs^2;4^	PFAs^3^
15–44	1.01	6.5	8.47
45–64	1.61	8.6	10.98
65+	2.85	12.4	14.3

Mean number	1.4	7.8	10.22

^1^ Non-frequent attenders

^2^ 1-year frequent attenders

^3^ persistent frequent attenders

^4^ All FAs of 2003 minus the 1yFAs that moved out of the practice or died.

In 2003, for patients of 15 years and older, 80% of all face-to-face consultations were with 37% of the registered patients. Another 37% of patients had not visited their GP at all during that year. In 2003, the 3,045 frequent attenders (10.6%) were responsible for 39% of all face-to-face consultations; the 470 persistent frequent attenders (1.6%) were responsible for 8% of all consultations.

### Morbidity

Table 2 shows the distribution of 9 medical problems or diagnoses across the three categories of non-frequent attenders and (persistent) frequent attenders. The most important findings are the high percentage of persistent frequent attenders with chronic somatic diseases (especially diabetes), psychological/psychiatric problems and MUPS and the substantial differences in morbidity for social and psychological/psychiatric problems, diabetes and MUPS. Persistent frequent attenders present with more medical problems (3.52) than 1-year frequent attenders (2.0) and non-frequent attenders (1.16). Age follows the predictable pattern of the older the patient, the more consultations and the more medical problems.

**Table 2:** Morbidity of non-frequent attenders, 1-year frequent attenders and persistent frequent attenders: prevalence and relative difference (non-frequent attenders 100)

	Non-Fas^1^	1yFAs^2;4^	PFAs^3^
	19120	2609	470

Diabetes mellitus	5.5	13.7 (250)	23.2 (421)

Chron. Cardiovasc. disease	13.7	23.4 (170)	37.7 (275)

Chron. resp. disease	9.8	17.8 (181)	27.2 (277)

(feelings of)anxiety	1.8	4.7 (261)	9.4 (522)

(feelings of) depression	3.2	6.4 (200)	8.7 (271)

Addictive behaviour	1.2	2.9 (241)	4.9 (408)

MUPS	6.8	13.1 (192)	25.3 (370)

Social problems	1.3	2.0 (153)	7.9 (607)

Psychological/psychiatric problems	9.2	20.6 (223)	37.0 (402)

Number of medical problems	1.16	2.00 (172)	3.52 (303)

^1 ^Non-frequent attenders

^2^ 1-year frequent attenders

^3^ persistent frequent attenders

^4^ All FAs of 2003 minus the 1yFAs that moved out of the practice or died.

Compared with both other groups, we see in persistent frequent attenders especially more social problems, more feelings of anxiety and more addictive behaviour. With the exception of diabetes these persistent frequent attenders differ less as far as the prevalence of chronic somatic diseases is concerned. In persistent frequent attenders, feelings of anxiety are more prevalent than feelings of depression. In 1-year frequent attenders, feelings of depression are more prevalent. (See Table 2)

### Number of prescriptions

Compared to both other groups, persistent frequent attenders received more prescriptions for anxiolytics and sleeping tablets, analgesics, antidepressants and antibiotics, Especially the high number of prescriptions for analgesics in persistent frequent attenders is remarkable (see Table 3).

**Table 3:** Mean number of prescriptions in non-frequent attenders, 1-year frequent attenders and persistent frequent attenders and relative difference (non-frequent attenders 100)

	Non-Fas^1^	1yFAs^2;4^	PFAs^3^
	19.120	2.609	470

Antibiotics	0.18	0.7 (388)	0.88 (488)

Analgesics	0.51	2.3 (457)	2.91 (570)

Anxiolytics	0.20	0.9 (450)	1.3 (650)

Hypnotics	0.19	0.7 (368)	0.99 (521)

Antidepressants	0.22	0.9 (409)	1.15 (523)

^1^Non-frequent attenders

^2 ^1-year frequent attenders

^3 ^persistent frequent attenders

^4 ^All FAs of 2003 minus the 1yFAs that moved out of the practice or died.

## Discussion

### Main findings

When analysing the consultations of all enlisted adult patients from 5 primary health centres during 3 consecutive years, we found that frequent attending is usually a self-limiting condition. One out every seven (15.4%) of patients who were a frequent attender in 2003 (or 18% of those frequent attenders who were enlisted for all three years) remained a frequent attender during two consecutive years. These persistent frequent attenders make up 1.6% of all enlisted patients of 15 years and older in 2003. GPs held about seven times more consultations with persistent frequent attenders compared with non-frequent attenders. Compared with both other groups, persistent frequent attenders presented more social problems, more psychiatric problems and MUPS, but also more chronic somatic diseases (especially diabetes). They received more prescriptions for psychotropic medication and analgesics.

### Study strength and limitations

An important strength of our study is the size and the longitudinal character of the dataset and the experience of the participating GPs. Most GPs have participated in the registration network for over 10 years and are used to accepting regular feedback on their registration activities. Prescriptions were extracted from the GPs' electronic medical record and the number of actual prescriptions is therefore reliable, although the amount of prescribed drugs is not. Prescription data in general practice are generally considered to be of higher quality than data on diagnoses [21]. As we used routinely collected data and did not plan any intervention in the normal practice routine, our data reflect the day-to-day business of general practice. Furthermore, the demographic data are accurate.

A limitation of our study, however, is that the data are restricted to "what the GP knows and registers". In particular, the problem lists could be inflated (if resolved problems are not removed) or subject to underreporting. Underreporting could be the case for patients with a low consultation frequency – thus inflating the contrast between frequent attenders en non-frequent attenders – and for patients who are relatively new in the practice. As the problem lists of all participating GPs are subject to evaluation on a regular basis, we think this problem is being dealt with as well as possible[22,23]. Many patients who suffer from an incurable disease become frequent attenders in the months prior to their death. Although our results may include terminally ill patients, only a few persistent frequent attenders were incurably sick and died soon after the study period (see Additional file 2). The GP practices in this study are situated in an urban area. This means that the results cannot be generalized and compared with practices in more rural areas. Unfortunately, socio-economic-level and ethnicity were not registered.

### Relevant literature

There is substantial literature about the characteristics and morbidity of 1-year frequent attenders. The few longitudinal studies show regression of attendance rates to the mean in the longer run [15,16,24,25]. However, studies on persistent frequent attendance used different definitions of frequent attendance and lacked the power to detect differences in morbidity and prescription data. Several trials have been conducted to test interventions for changing consultation behaviour and/or morbidity of frequent attenders [12]. Only one study consisting of 2 randomized clinical trials used frequent attendance over a period of 2 years [13,14]. All others included 1-year frequent attenders [26-28]. Although no study found evidence to support the possibility that healthcare utilization of frequent attenders can be influenced, the study that included frequent attenders for two years did find evidence that treatment of major depressive disorder in a subgroup of depressed frequent attenders improved the patients' symptoms and quality of life. Unfortunately an effort to develop a rule to predict, with readily available indicators from GPs electronic medical record, future persisting frequent attendance did only succeeded modestly[18].

### Implications for future research or clinical practice

Knowing that frequent attendance is predominantly a temporary phenomenon and because of the continuous high workload, the high prevalence of diseases and the considerable use of medication, we think that only persistent frequent attenders deserve further attention. Regarding the important role of psychiatric problems (especially anxiety), social problems and MUPS in persisting frequent attendance and regarding the already existing intensive disease management programs for chronic somatic diseases, it seems logical to focus on social and psychiatric problems and MUPS of frequent attenders in order to try to improve their quality of life and to prevent the continuation of frequent consulting behaviour.

## Conclusion

We conclude that, compared with normal attenders, 1-year frequent attenders have many somatic and psychiatric problems and are prescribed much medication. They constitute a substantial part of the clinical work of a GP. One out of every seven 1-year frequent attenders persists to consult frequently during a period of two consecutive years. Compared to 1-year frequent attenders, persistent frequent attenders have even more consultations with their GP, suffer not only from more somatic diseases but especially from more social and psychiatric problems and MUPS and are prescribed more (psychotropic and analgesic) medication.

## Competing interests

The authors declare that they have no competing interests.

## Authors' contributions

FS conceived the study, participated in its design and coordination and drafted the final manuscript. HB participated in the design of the study, performed the statistical analysis and helped to draft the manuscript. GtR participated in the design, helped to perform the statistical analysis and helped to draft the manuscript. HvW helped to conceive the study, participated in its design and helped to draft the manuscript. All authors read, commented upon and approved the final manuscript.

## Pre-publication history

The pre-publication history for this paper can be accessed here:



## Supplementary Material

Additional file 1 **Appendix.** Selected problems and diseases with ICPC-code. Click here for file

Additional file 2 **Box 1.** Multivariable analysis. Click here for file
