# Supplementary material for: Epidemiology of frequent attenders: a 3-year historic cohort study comparing attendance, morbidity and prescriptions of one-year and persistent frequent attenders
Source: BMC Public Health. 2009 Jan 24;9:36. doi: 10.1186/1471-2458-9-36 (PMC2649070; doi:10.1186/1471-2458-9-36)
Supplement: Additional file 1 — Appendix. Selected problems and diseases with ICPC-code. [file 1471-2458-9-36-S1.doc]

Addional file 2:

**Appendix: Selected problems and diseases with ICPC-code**

| Medical problem | Prevalence at end of first year (n/1000, ≥ 15 years of age) | ICPC code | Problem |
| --- | --- | --- | --- |
| Diabetes | 60,3 | T90 | Diabetes mellitus type 1 and 2 |
| Chronic Cardiovascular disease | 14,6  10,5  6,1  6,6  8,1  0,1  7,6  78,6  16,8  5,5  10,2  1,6  8,1 | K74  K75  K76  K77  K78  K82  K83  K86  K87  K89  K90  K91  K92 | Angina pectoris  Acute myocardial infarction  Other and chronic. ischemic heart disease  Heart failure  Atrial fibrillation/flutter  Pulmonary heart disease  Heart valve dis. NOS, non-rheum.  Hypertension, uncomplicated  Hypertension with involve. target organs  Transient cerebral ischemia  Cerebrovascular accident; stroke  Atherosclerosis excl. heart/brain  Other arter. Obstr. / periph. vascular disease |
| Chronic respiratory problems | 0,7  2,4  16,8  40,9  49,3 | R70  R91  R95  R96  R97 | Tuberculosis resp  Chronic bronchitis/ bronchiectasis  Emphysema/COPD  Astma  Allergic rhinitis ; Hay fever |
| Psychological/Psychiatric problems   1. Anxious feeling 2. depressed feeling     3) addictive behaviour | 109,7  5,8  11,0  0,3  0,7  0,8  0  0  0,9  0  0  0,2  0  0  0  0,2  2,1  29,0  8,1  0,5  1,0  1,9  3,2 | All P  P01  P74  P09  A-Y27  A-D26  L26  N26  R-Y26  A25  B25  K24  K25  X23/Y25  X24/Y24  X25  P03  P76  P15  P16  P17  P18  P19 | All P-codes  feeling anxious/nervous/tense  Anxiety disorder/anxiety state  Concern sexual preference  Fear of other disease of….  Fear of cancer of….  Fear of cancer locom. tract  Fear of cancer neuro system  Fear of cancer …. system  Fear of death  Fear of AIDS  Fear of heart attack  Fear of hypertension  Fear of venereal disease  Fear of sexual dysfunction  Fear of genital cancer  Feeling depressed  Depressive disorder  Chronic alcohol abuse  Acute alcohol abuse  Tobacco abuse  Medicinal abuse  Drug abuse |
| Social problems | 15,6 | All Z | All socio-economic problems |
| Medically unexplained physical problems (MUPS) | 4,1    2,7  18,0  1,8  0,8  3,9  4,8  2,3  2,1  2,1  0  0  0,3  0  0,4  0,5  2,3  0,5  1,1  0  0,4  0  3,4  1,9  10,7 | L01  L02  L03  L04  L18  N01  N02  A04  P06  P20  T03  T07  T08  P04  R02  K04  N17  R21  N06  D09  D11  D08  D12  D01  D93 | Neck sympt. /complaints (excl. headache)  Back symptoms/complaints  Low back compl. w/o radiation  Chest symptoms/complaints  Muscle pain / fibrositis  Headache  Tension headache  General weakness/tiredness  Disturbance of sleep/insomnia  Disturbance memory, concentration  Loss of appetite  Weight gain  Weight loss  Feeling/behaving irritable  Shortness of breath, dyspnoea  Palpitations/ aware of heartbeat  Vertigo/Dizziness  Symptoms / compl. throat  Other sens. distur. / ab invol.mov.  Nausea  Diarrhoea / Loose bowels  Flatulence/gas pain/belching  Constipation  Generalized Abdominal pain/cramps  Irritable bowel syndrome |
