# Supplementary material for: Epidemiology of frequent attenders: a 3-year historic cohort study comparing attendance, morbidity and prescriptions of one-year and persistent frequent attenders
Source: BMC Public Health. 2009 Jan 24;9:36. doi: 10.1186/1471-2458-9-36 (PMC2649070; doi:10.1186/1471-2458-9-36)
Supplement: Additional file 2 — Box 1. Multivariable analysis. [file 1471-2458-9-36-S2.doc]

**Multivariable analysis**

*Loss to follow-up*

The numbers of frequent attenders found were as follows: 2003, 3,045 (10.6%); 2004, 2,897 (10.2%); 2005, 2,499 (9.3%). 368 patients (12%) were lost at some point over the two years of follow-up. We argued that, in theory, a potential frequent attender might move out of the practice due to dissatisfaction with care. The resulting selection bias may attenuate associations found between the selected indicators and frequent attendance. We tested our hypothesis in a multivariable logistic regression analysis with an indicator variable “1 = moved house” and “0 otherwise” as the dependent variable and 9 independent indicators. Our hypothesis was not confirmed. On the contrary, we found some evidence that those with at least one chronic somatic illness were less likely to have moved out of the practice (odds ratio 0.73 (95%CI from 0.54 to 0.99)); all other associations were neither strong nor significant. These results support the view that important selection bias (due to moving out of the practice) is unlikely. Seventy-one patients had died over the two year follow-up period. To assess the extent to which these deaths caused selection bias (informative censoring), we performed a sensitivity analysis: (1) we repaired the entire cohort using inverse probability weighting, where the weights were derived after fitting a logistic regression model with death as the dependent variable and (2) we assumed that the 71 deaths were all persisting frequent attenders.[29] The resulting statistics for these two additional analyses were very close to the results from the analysis in which we assumed that those who died would not become a persisting frequent attender. Specifically, the AUCs for the additional two analyses were 0.662 (0.635 to 0.688), and 0.672 (0.646 to 0.698), respectively. To determine whether frequent attending is a sign of terminal disease we checked how many of our persistent frequent attenders died in the years after our analysis: Of the 470 persistent frequent attenders 6 died in 2006 (1%) and 10 in 2007(2.1%).
